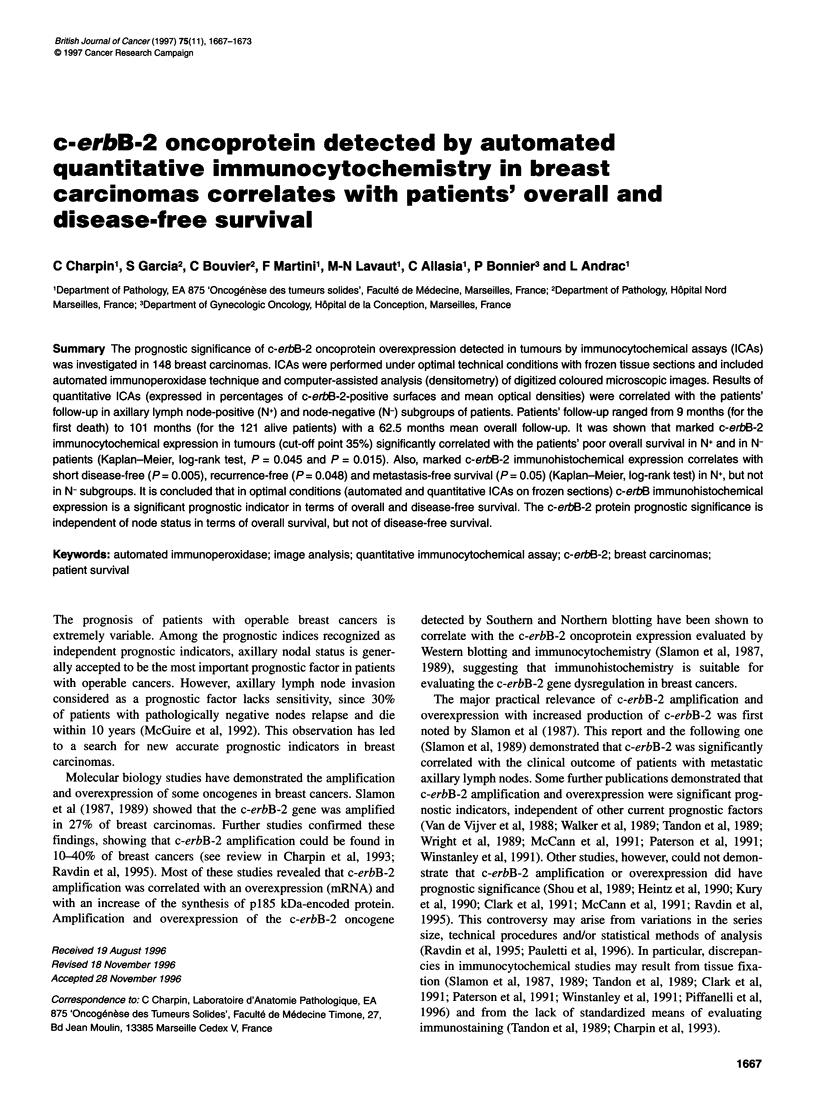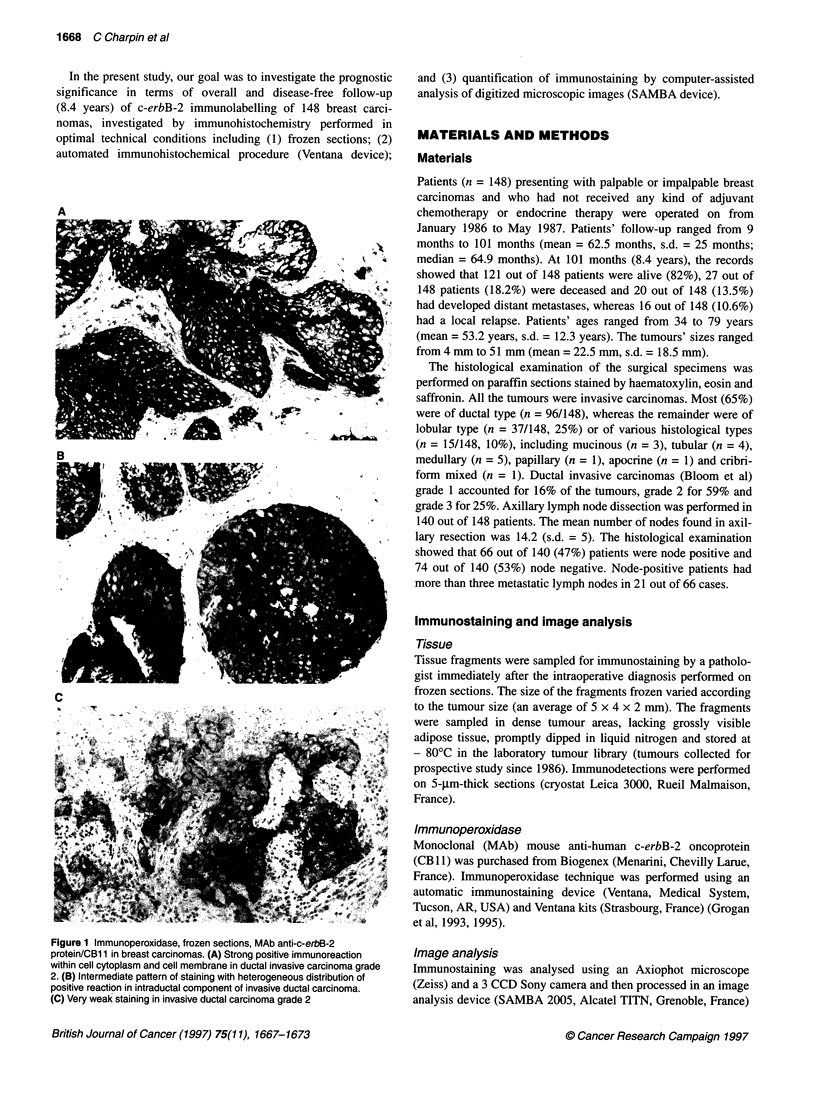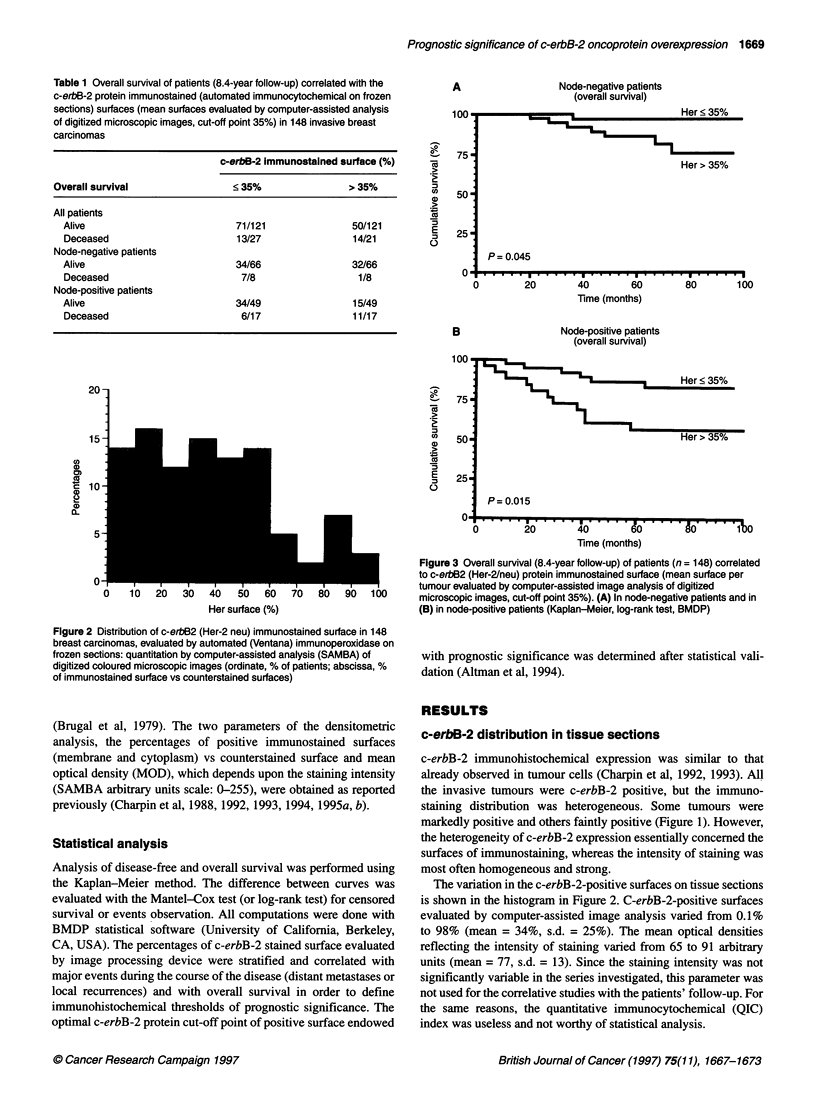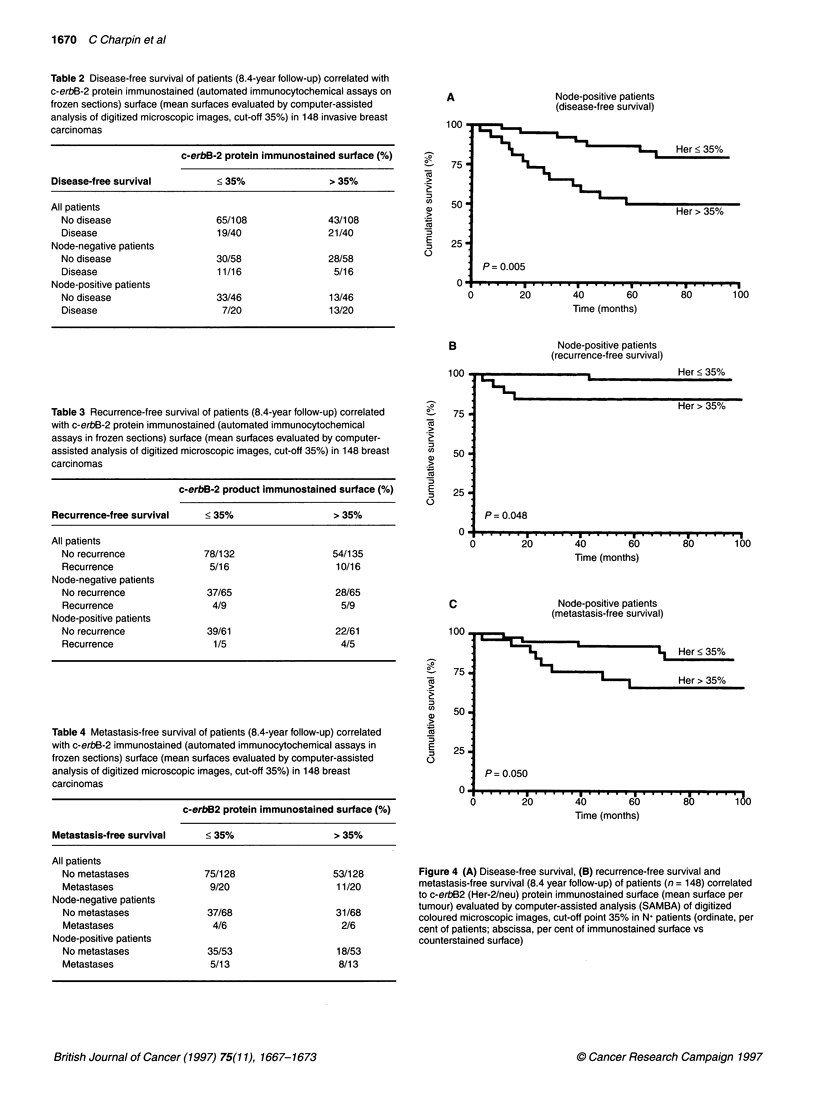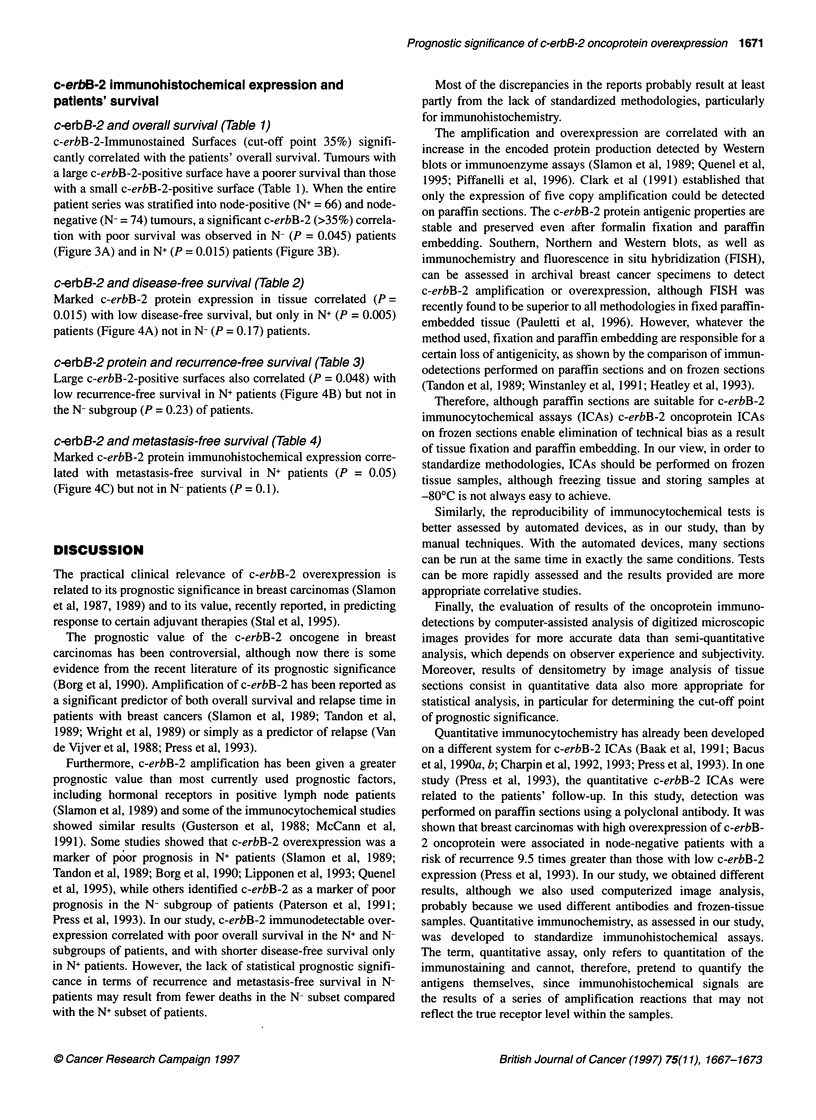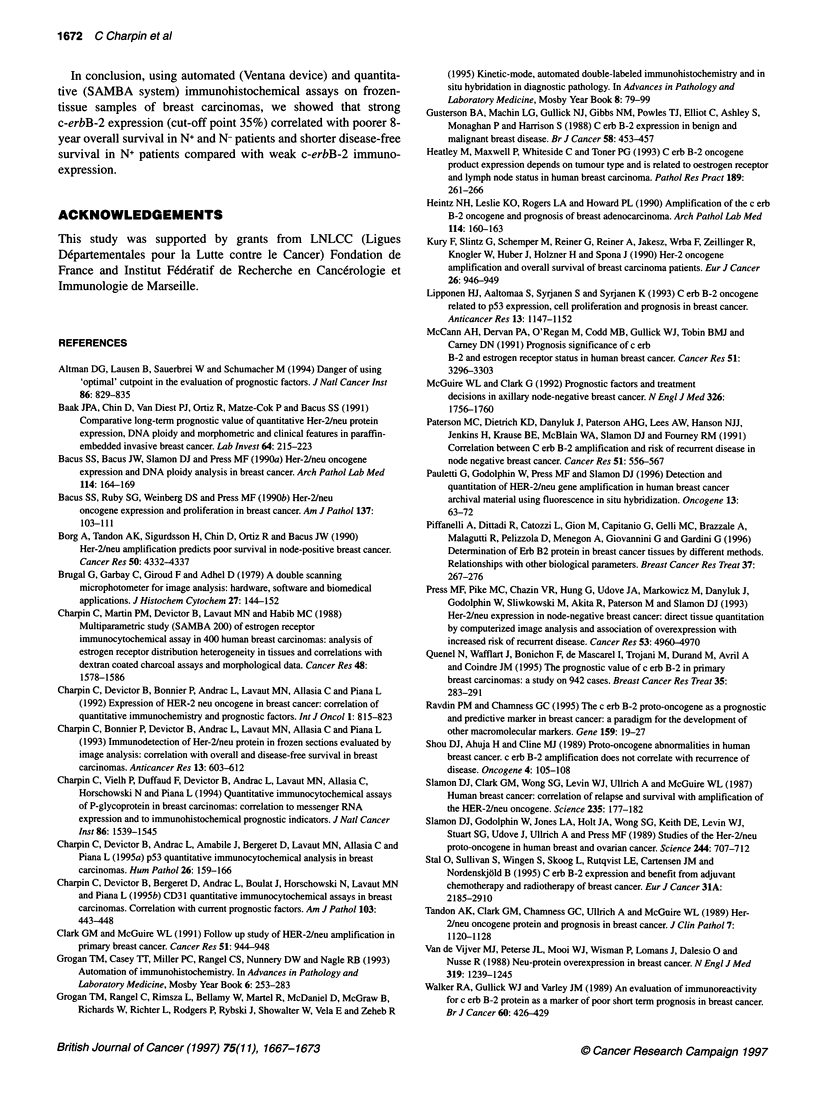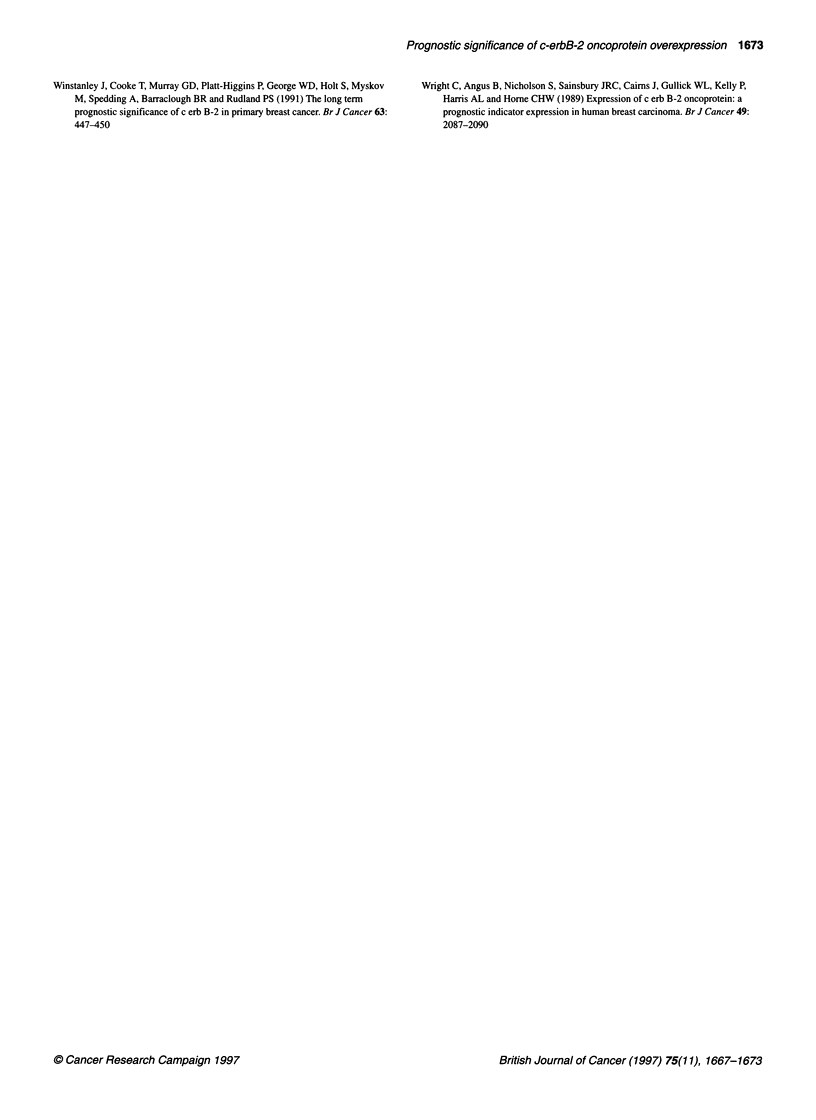# c-erbB-2 oncoprotein detected by automated quantitative immunocytochemistry in breast carcinomas correlates with patients' overall and disease-free survival.

**DOI:** 10.1038/bjc.1997.283

**Published:** 1997

**Authors:** C. Charpin, S. Garcia, C. Bouvier, F. Martini, M. N. Lavaut, C. Allasia, P. Bonnier, L. Andrac

**Affiliations:** Department of Pathology, EA 875 OncogÃ©nÃ¨se des tumeurs solides, FacultÃ© de MÃ©decine, Marseilles, France.

## Abstract

**Images:**


British Journal of Cancer (1997) 75(11), 1667–1673
© 1997 Cancer Research Campaign

### c-*erb*B-2 oncoprotein detected by automated quantitative immunocytochemistry in breast carcinomas correlates with patients' overall and disease-free survival

**C Charpin[1], S Garcia[2], C Bouvier[2], F Martini[1], M-N Lavaut[1], C Allasia[1], P Bonnier[3] and L Andrac[1]**

[1]Department of Pathology, EA 875 'Oncogénèse des tumeurs solides', Faculté de Médecine, Marseilles, France; [2]Department of Pathology, Hôpital Nord Marseilles, France; [3]Department of Gynecologic Oncology, Hôpital de la Conception, Marseilles, France

**Summary** The prognostic significance of c-*erb*B-2 oncoprotein overexpression detected in tumours by immunocytochemical assays (ICAs) was investigated in 148 breast carcinomas. ICAs were performed under optimal technical conditions with frozen tissue sections and included automated immunoperoxidase technique and computer-assisted analysis (densitometry) of digitized coloured microscopic images. Results of quantitative ICAs (expressed in percentages of c-*erb*B-2-positive surfaces and mean optical densities) were correlated with the patients' follow-up in axillary lymph node-positive ($N^+$) and node-negative ($N^-$) subgroups of patients. Patients' follow-up ranged from 9 months (for the first death) to 101 months (for the 121 alive patients) with a 62.5 months mean overall follow-up. It was shown that marked c-*erb*B-2 immunocytochemical expression in tumours (cut-off point 35%) significantly correlated with the patients' poor overall survival in $N^+$ and in $N^-$ patients (Kaplan–Meier, log-rank test, $P$ = 0.045 and $P$ = 0.015). Also, marked c-*erb*B-2 immunohistochemical expression correlates with short disease-free ($P$ = 0.005), recurrence-free ($P$ = 0.048) and metastasis-free survival ($P$ = 0.05) (Kaplan–Meier, log-rank test) in $N^+$, but not in $N^-$ subgroups. It is concluded that in optimal conditions (automated and quantitative ICAs on frozen sections) c-*erb*B immunohistochemical expression is a significant prognostic indicator in terms of overall and disease-free survival. The c-*erb*B-2 protein prognostic significance is independent of node status in terms of overall survival, but not of disease-free survival.

**Keywords:** automated immunoperoxidase; image analysis; quantitative immunocytochemical assay; c-*erb*B-2; breast carcinomas; patient survival

The prognosis of patients with operable breast cancers is extremely variable. Among the prognostic indices recognized as independent prognostic indicators, axillary nodal status is generally accepted to be the most important prognostic factor in patients with operable cancers. However, axillary lymph node invasion considered as a prognostic factor lacks sensitivity, since 30% of patients with pathologically negative nodes relapse and die within 10 years (McGuire et al, 1992). This observation has led to a search for new accurate prognostic indicators in breast carcinomas.

Molecular biology studies have demonstrated the amplification and overexpression of some oncogenes in breast cancers. Slamon et al (1987, 1989) showed that the c-*erb*B-2 gene was amplified in 27% of breast carcinomas. Further studies confirmed these findings, showing that c-*erb*B-2 amplification could be found in 10–40% of breast cancers (see review in Charpin et al, 1993; Ravdin et al, 1995). Most of these studies revealed that c-*erb*B-2 amplification was correlated with an overexpression (mRNA) and with an increase of the synthesis of p185 kDa-encoded protein. Amplification and overexpression of the c-*erb*B-2 oncogene detected by Southern and Northern blotting have been shown to correlate with the c-*erb*B-2 oncoprotein expression evaluated by Western blotting and immunocytochemistry (Slamon et al, 1987, 1989), suggesting that immunohistochemistry is suitable for evaluating the c-*erb*B-2 gene dysregulation in breast cancers.

The major practical relevance of c-*erb*B-2 amplification and overexpression with increased production of c-*erb*B-2 was first noted by Slamon et al (1987). This report and the following one (Slamon et al, 1989) demonstrated that c-*erb*B-2 was significantly correlated with the clinical outcome of patients with metastatic axillary lymph nodes. Some further publications demonstrated that c-*erb*B-2 amplification and overexpression were significant prognostic indicators, independent of other current prognostic factors (Van de Vijver et al, 1988; Walker et al, 1989; Tandon et al, 1989; Wright et al, 1989; McCann et al, 1991; Paterson et al, 1991; Winstanley et al, 1991). Other studies, however, could not demonstrate that c-*erb*B-2 amplification or overexpression did have prognostic significance (Shou et al, 1989; Heintz et al, 1990; Kury et al, 1990; Clark et al, 1991; McCann et al, 1991; Ravdin et al, 1995). This controversy may arise from variations in the series size, technical procedures and/or statistical methods of analysis (Ravdin et al, 1995; Pauletti et al, 1996). In particular, discrepancies in immunocytochemical studies may result from tissue fixation (Slamon et al, 1987, 1989; Tandon et al, 1989; Clark et al, 1991; Paterson et al, 1991; Winstanley et al, 1991; Piffanelli et al, 1996) and from the lack of standardized means of evaluating immunostaining (Tandon et al, 1989; Charpin et al, 1993).

*Received 19 August 1996*
*Revised 18 November 1996*
*Accepted 28 November 1996*

*Correspondence to:* C Charpin, Laboratoire d'Anatomie Pathologique, EA 875 'Oncogénèse des Tumeurs Solides', Faculté de Médecine Timone, 27, Bd Jean Moulin, 13385 Marseille Cedex V, France

In the present study, our goal was to investigate the prognostic significance in terms of overall and disease-free follow-up (8.4 years) of c-*erb*B-2 immunolabelling of 148 breast carcinomas, investigated by immunohistochemistry performed in optimal technical conditions including (1) frozen sections; (2) automated immunohistochemical procedure (Ventana device); and (3) quantification of immunostaining by computer-assisted analysis of digitized microscopic images (SAMBA device).

**Figure 1** Immunoperoxidase, frozen sections, MAb anti-c-*erb*B-2 protein/CB11 in breast carcinomas. **(A)** Strong positive immunoreaction within cell cytoplasm and cell membrane in ductal invasive carcinoma grade 2. **(B)** Intermediate pattern of staining with heterogeneous distribution of positive reaction in intraductal component of invasive ductal carcinoma. **(C)** Very weak staining in invasive ductal carcinoma grade 2

### MATERIALS AND METHODS

### Materials

Patients ($n$ = 148) presenting with palpable or impalpable breast carcinomas and who had not received any kind of adjuvant chemotherapy or endocrine therapy were operated on from January 1986 to May 1987. Patients' follow-up ranged from 9 months to 101 months (mean = 62.5 months, s.d. = 25 months; median = 64.9 months). At 101 months (8.4 years), the records showed that 121 out of 148 patients were alive (82%), 27 out of 148 patients (18.2%) were deceased and 20 out of 148 (13.5%) had developed distant metastases, whereas 16 out of 148 (10.6%) had a local relapse. Patients' ages ranged from 34 to 79 years (mean = 53.2 years, s.d. = 12.3 years). The tumours' sizes ranged from 4 mm to 51 mm (mean = 22.5 mm, s.d. = 18.5 mm).

The histological examination of the surgical specimens was performed on paraffin sections stained by haematoxylin, eosin and saffronin. All the tumours were invasive carcinomas. Most (65%) were of ductal type ($n$ = 96/148), whereas the remainder were of lobular type ($n$ = 37/148, 25%) or of various histological types ($n$ = 15/148, 10%), including mucinous ($n$ = 3), tubular ($n$ = 4), medullary ($n$ = 5), papillary ($n$ = 1), apocrine ($n$ = 1) and cribriform mixed ($n$ = 1). Ductal invasive carcinomas (Bloom et al) grade 1 accounted for 16% of the tumours, grade 2 for 59% and grade 3 for 25%. Axillary lymph node dissection was performed in 140 out of 148 patients. The mean number of nodes found in axillary resection was 14.2 (s.d. = 5). The histological examination showed that 66 out of 140 (47%) patients were node positive and 74 out of 140 (53%) node negative. Node-positive patients had more than three metastatic lymph nodes in 21 out of 66 cases.

### Immunostaining and image analysis

*Tissue*

Tissue fragments were sampled for immunostaining by a pathologist immediately after the intraoperative diagnosis performed on frozen sections. The size of the fragments frozen varied according to the tumour size (an average of 5 × 4 × 2 mm). The fragments were sampled in dense tumour areas, lacking grossly visible adipose tissue, promptly dipped in liquid nitrogen and stored at – 80°C in the laboratory tumour library (tumours collected for prospective study since 1986). Immunodetections were performed on 5-μm-thick sections (cryostat Leica 3000, Rueil Malmaison, France).

*Immunoperoxidase*

Monoclonal (MAb) mouse anti-human c-*erb*B-2 oncoprotein (CB11) was purchased from Biogenex (Menarini, Chevilly Larue, France). Immunoperoxidase technique was performed using an automatic immunostaining device (Ventana, Medical System, Tucson, AR, USA) and Ventana kits (Strasbourg, France) (Grogan et al, 1993, 1995).

*Image analysis*

Immunostaining was analysed using an Axiophot microscope (Zeiss) and a 3 CCD Sony camera and then processed in an image analysis device (SAMBA 2005, Alcatel TITN, Grenoble, France)

 © Cancer Research Campaign 1997

**Table 1** Overall survival of patients (8.4-year follow-up) correlated with the c-*erb*B-2 protein immunostained (automated immunocytochemical on frozen sections) surfaces (mean surfaces evaluated by computer-assisted analysis of digitized microscopic images, cut-off point 35%) in 148 invasive breast carcinomas

| | c-*erb*B-2 immunostained surface (%) | |
|---|---|---|
| **Overall survival** | **≤ 35%** | **> 35%** |
| All patients | | |
| Alive | 71/121 | 50/121 |
| Deceased | 13/27 | 14/21 |
| Node-negative patients | | |
| Alive | 34/66 | 32/66 |
| Deceased | 7/8 | 1/8 |
| Node-positive patients | | |
| Alive | 34/49 | 15/49 |
| Deceased | 6/17 | 11/17 |

**Figure 2** Distribution of c-*erb*B2 (Her-2 neu) immunostained surface in 148 breast carcinomas, evaluated by automated (Ventana) immunoperoxidase on frozen sections: quantitation by computer-assisted analysis (SAMBA) of digitized coloured microscopic images (ordinate, % of patients; abscissa, % of immunostained surface vs counterstained surfaces)

**Figure 3** Overall survival (8.4-year follow-up) of patients ($n$ = 148) correlated to c-*erb*B2 (Her-2/neu) protein immunostained surface (mean surface per tumour evaluated by computer-assisted image analysis of digitized microscopic images, cut-off point 35%). **(A)** In node-negative patients and in **(B)** in node-positive patients (Kaplan–Meier, log-rank test, BMDP)

(Brugal et al, 1979). The two parameters of the densitometric analysis, the percentages of positive immunostained surfaces (membrane and cytoplasm) vs counterstained surface and mean optical density (MOD), which depends upon the staining intensity (SAMBA arbitrary units scale: 0–255), were obtained as reported previously (Charpin et al, 1988, 1992, 1993, 1994, 1995*a*, *b*).

### Statistical analysis

Analysis of disease-free and overall survival was performed using the Kaplan–Meier method. The difference between curves was evaluated with the Mantel–Cox test (or log-rank test) for censored survival or events observation. All computations were done with BMDP statistical software (University of California, Berkeley, CA, USA). The percentages of c-*erb*B-2 stained surface evaluated by image processing device were stratified and correlated with major events during the course of the disease (distant metastases or local recurrences) and with overall survival in order to define immunohistochemical thresholds of prognostic significance. The optimal c-*erb*B-2 protein cut-off point of positive surface endowed with prognostic significance was determined after statistical validation (Altman et al, 1994).

### RESULTS

### c-*erb*B-2 distribution in tissue sections c-*erb*B-2 immunohistochemical expression was similar to that already observed in tumour cells (Charpin et al, 1992, 1993). All the invasive tumours were c-*erb*B-2 positive, but the immunostaining distribution was heterogeneous. Some tumours were markedly positive and others faintly positive (Figure 1). However, the heterogeneity of c-*erb*B-2 expression essentially concerned the surfaces of immunostaining, whereas the intensity of staining was most often homogeneous and strong.

The variation in the c-*erb*B-2-positive surfaces on tissue sections is shown in the histogram in Figure 2. C-*erb*B-2-positive surfaces evaluated by computer-assisted image analysis varied from 0.1% to 98% (mean = 34%, s.d. = 25%). The mean optical densities reflecting the intensity of staining varied from 65 to 91 arbitrary units (mean = 77, s.d. = 13). Since the staining intensity was not significantly variable in the series investigated, this parameter was not used for the correlative studies with the patients' follow-up. For the same reasons, the quantitative immunocytochemical (QIC) index was useless and not worthy of statistical analysis.

© Cancer Research Campaign 1997

**Table 2** Disease-free survival of patients (8.4-year follow-up) correlated with c-*erb*B-2 protein immunostained (automated immunocytochemical assays on frozen sections) surface (mean surfaces evaluated by computer-assisted analysis of digitized microscopic images, cut-off 35%) in 148 invasive breast carcinomas

| | c-*erb*B-2 protein immunostained surface (%) | |
|---|---|---|
| **Disease-free survival** | **≤ 35%** | **> 35%** |
| All patients | | |
| No disease | 65/108 | 43/108 |
| Disease | 19/40 | 21/40 |
| Node-negative patients | | |
| No disease | 30/58 | 28/58 |
| Disease | 11/16 | 5/16 |
| Node-positive patients | | |
| No disease | 33/46 | 13/46 |
| Disease | 7/20 | 13/20 |

**Table 3** Recurrence-free survival of patients (8.4-year follow-up) correlated with c-*erb*B-2 protein immunostained (automated immunocytochemical assays in frozen sections) surface (mean surfaces evaluated by computer-assisted analysis of digitized microscopic images, cut-off 35%) in 148 breast carcinomas

| | c-*erb*B-2 product immunostained surface (%) | |
|---|---|---|
| **Recurrence-free survival** | **≤ 35%** | **> 35%** |
| All patients | | |
| No recurrence | 78/132 | 54/135 |
| Recurrence | 5/16 | 10/16 |
| Node-negative patients | | |
| No recurrence | 37/65 | 28/65 |
| Recurrence | 4/9 | 5/9 |
| Node-positive patients | | |
| No recurrence | 39/61 | 22/61 |
| Recurrence | 1/5 | 4/5 |

**Table 4** Metastasis-free survival of patients (8.4-year follow-up) correlated with c-*erb*B-2 immunostained (automated immunocytochemical assays in frozen sections) surface (mean surfaces evaluated by computer-assisted analysis of digitized microscopic images, cut-off 35%) in 148 breast carcinomas

| | c-*erb*B2 protein immunostained surface (%) | |
|---|---|---|
| **Metastasis-free survival** | **≤ 35%** | **> 35%** |
| All patients | | |
| No metastases | 75/128 | 53/128 |
| Metastases | 9/20 | 11/20 |
| Node-negative patients | | |
| No metastases | 37/68 | 31/68 |
| Metastases | 4/6 | 2/6 |
| Node-positive patients | | |
| No metastases | 35/53 | 18/53 |
| Metastases | 5/13 | 8/13 |

**Figure 4** **(A)** Disease-free survival, **(B)** recurrence-free survival and metastasis-free survival (8.4 year follow-up) of patients ($n$ = 148) correlated to c-*erb*B2 (Her-2/neu) protein immunostained surface (mean surface per tumour) evaluated by computer-assisted analysis (SAMBA) of digitized coloured microscopic images, cut-off point 35% in N+ patients (ordinate, per cent of patients; abscissa, per cent of immunostained surface vs counterstained surface)

© Cancer Research Campaign 1997

### c-*erb*B-2 immunohistochemical expression and patients' survival

*c-*erb*B-2 and overall survival (Table 1)*

c-*erb*B-2-Immunostained Surfaces (cut-off point 35%) significantly correlated with the patients' overall survival. Tumours with a large c-*erb*B-2-positive surface have a poorer survival than those with a small c-*erb*B-2-positive surface (Table 1). When the entire patient series was stratified into node-positive ($N^+ = 66$) and node-negative ($N^- = 74$) tumours, a significant c-*erb*B-2 (>35%) correlation with poor survival was observed in $N^-$ ($P = 0.045$) patients (Figure 3A) and in $N^+$ ($P = 0.015$) patients (Figure 3B).

*c-*erb*B-2 and disease-free survival (Table 2)*

Marked c-*erb*B-2 protein expression in tissue correlated ($P = 0.015$) with low disease-free survival, but only in $N^+$ ($P = 0.005$) patients (Figure 4A) not in $N^-$ ($P = 0.17$) patients.

*c-*erb*B-2 protein and recurrence-free survival (Table 3)*

Large c-*erb*B-2-positive surfaces also correlated ($P = 0.048$) with low recurrence-free survival in $N^+$ patients (Figure 4B) but not in the $N^-$ subgroup ($P = 0.23$) of patients.

*c-*erb*B-2 and metastasis-free survival (Table 4)*

Marked c-*erb*B-2 protein immunohistochemical expression correlated with metastasis-free survival in $N^+$ patients ($P = 0.05$) (Figure 4C) but not in $N^-$ patients ($P = 0.1$).

### DISCUSSION

The practical clinical relevance of c-*erb*B-2 overexpression is related to its prognostic significance in breast carcinomas (Slamon et al, 1987, 1989) and to its value, recently reported, in predicting response to certain adjuvant therapies (Stal et al, 1995).

The prognostic value of the c-*erb*B-2 oncogene in breast carcinomas has been controversial, although now there is some evidence from the recent literature of its prognostic significance (Borg et al, 1990). Amplification of c-*erb*B-2 has been reported as a significant predictor of both overall survival and relapse time in patients with breast cancers (Slamon et al, 1989; Tandon et al, 1989; Wright et al, 1989) or simply as a predictor of relapse (Van de Vijver et al, 1988; Press et al, 1993).

Furthermore, c-*erb*B-2 amplification has been given a greater prognostic value than most currently used prognostic factors, including hormonal receptors in positive lymph node patients (Slamon et al, 1989) and some of the immunocytochemical studies showed similar results (Gusterson et al, 1988; McCann et al, 1991). Some studies showed that c-*erb*B-2 overexpression was a marker of poor prognosis in $N^+$ patients (Slamon et al, 1989; Tandon et al, 1989; Borg et al, 1990; Lipponen et al, 1993; Quenel et al, 1995), while others identified c-*erb*B-2 as a marker of poor prognosis in the $N^-$ subgroup of patients (Paterson et al, 1991; Press et al, 1993). In our study, c-*erb*B-2 immunodetectable overexpression correlated with poor overall survival in the $N^+$ and $N^-$ subgroups of patients, and with shorter disease-free survival only in $N^+$ patients. However, the lack of statistical prognostic significance in terms of recurrence and metastasis-free survival in $N^-$ patients may result from fewer deaths in the $N^-$ subset compared with the $N^+$ subset of patients.

Most of the discrepancies in the reports probably result at least partly from the lack of standardized methodologies, particularly for immunohistochemistry.

The amplification and overexpression are correlated with an increase in the encoded protein production detected by Western blots or immunoenzyme assays (Slamon et al, 1989; Quenel et al, 1995; Piffanelli et al, 1996). Clark et al (1991) established that only the expression of five copy amplification could be detected on paraffin sections. The c-*erb*B-2 protein antigenic properties are stable and preserved even after formalin fixation and paraffin embedding. Southern, Northern and Western blots, as well as immunochemistry and fluorescence in situ hybridization (FISH), can be assessed in archival breast cancer specimens to detect c-*erb*B-2 amplification or overexpression, although FISH was recently found to be superior to all methodologies in fixed paraffin-embedded tissue (Pauletti et al, 1996). However, whatever the method used, fixation and paraffin embedding are responsible for a certain loss of antigenicity, as shown by the comparison of immunodetections performed on paraffin sections and on frozen sections (Tandon et al, 1989; Winstanley et al, 1991; Heatley et al, 1993).

Therefore, although paraffin sections are suitable for c-*erb*B-2 immunocytochemical assays (ICAs) c-*erb*B-2 oncoprotein ICAs on frozen sections enable elimination of technical bias as a result of tissue fixation and paraffin embedding. In our view, in order to standardize methodologies, ICAs should be performed on frozen tissue samples, although freezing tissue and storing samples at –80°C is not always easy to achieve.

Similarly, the reproducibility of immunocytochemical tests is better assessed by automated devices, as in our study, than by manual techniques. With the automated devices, many sections can be run at the same time in exactly the same conditions. Tests can be more rapidly assessed and the results provided are more appropriate correlative studies.

Finally, the evaluation of results of the oncoprotein immunodetections by computer-assisted analysis of digitized microscopic images provides for more accurate data than semi-quantitative analysis, which depends on observer experience and subjectivity. Moreover, results of densitometry by image analysis of tissue sections consist in quantitative data also more appropriate for statistical analysis, in particular for determining the cut-off point of prognostic significance.

Quantitative immunocytochemistry has already been developed on a different system for c-*erb*B-2 ICAs (Baak et al, 1991; Bacus et al, 1990*a*, *b*; Charpin et al, 1992, 1993; Press et al, 1993). In one study (Press et al, 1993), the quantitative c-*erb*B-2 ICAs were related to the patients' follow-up. In this study, detection was performed on paraffin sections using a polyclonal antibody. It was shown that breast carcinomas with high overexpression of c-*erb*B-2 oncoprotein were associated in node-negative patients with a risk of recurrence 9.5 times greater than those with low c-*erb*B-2 expression (Press et al, 1993). In our study, we obtained different results, although we also used computerized image analysis, probably because we used different antibodies and frozen-tissue samples. Quantitative immunochemistry, as assessed in our study, was developed to standardize immunohistochemical assays. The term, quantitative assay, only refers to quantitation of the immunostaining and cannot, therefore, pretend to quantify the antigens themselves, since immunohistochemical signals are the results of a series of amplification reactions that may not reflect the true receptor level within the samples.

© Cancer Research Campaign 1997

In conclusion, using automated (Ventana device) and quantitative (SAMBA system) immunohistochemical assays on frozen-tissue samples of breast carcinomas, we showed that strong c-*erb*B-2 expression (cut-off point 35%) correlated with poorer 8-year overall survival in $N^+$ and $N^-$ patients and shorter disease-free survival in $N^+$ patients compared with weak c-*erb*B-2 immunoexpression.

### ACKNOWLEDGEMENTS

This study was supported by grants from LNLCC (Ligues Départementales pour la Lutte contre le Cancer) Fondation de France and Institut Fédératif de Recherche en Cancérologie et Immunologie de Marseille.

### REFERENCES

Altman DG, Lausen B, Sauerbrei W and Schumacher M (1994) Danger of using 'optimal' cutpoint in the evaluation of prognostic factors. *J Natl Cancer Inst* **86**: 829–835

Baak JPA, Chin D, Van Diest PJ, Ortiz R, Matze-Cok P and Bacus SS (1991) Comparative long-term prognostic value of quantitative Her-2/neu protein expression, DNA ploidy and morphometric and clinical features in paraffin-embedded invasive breast cancer. *Lab Invest* **64**: 215–223

Bacus SS, Bacus JW, Slamon DJ and Press MF (1990*a*) Her-2/neu oncogene expression and DNA ploidy analysis in breast cancer. *Arch Pathol Lab Med* **114**: 164–169

Bacus SS, Ruby SG, Weinberg DS and Press MF (1990*b*) Her-2/neu oncogene expression and proliferation in breast cancer. *Am J Pathol* **137**: 103–111

Borg A, Tandon AK, Sigurdsson H, Chin D, Ortiz R and Bacus JW (1990) Her-2/neu amplification predicts poor survival in node-positive breast cancer. *Cancer Res* **50**: 4332–4337

Brugal G, Garbay C, Giroud F and Adhel D (1979) A double scanning microphotometer for image analysis: hardware, software and biomedical applications. *J Histochem Cytochem* **27**: 144–152

Charpin C, Martin PM, Devictor B, Lavaut MN and Habib MC (1988) Multiparametric study (SAMBA 200) of estrogen receptor immunocytochemical assay in 400 human breast carcinomas: analysis of estrogen receptor distribution heterogeneity in tissues and correlations with dextran coated charcoal assays and morphological data. *Cancer Res* **48**: 1578–1586

Charpin C, Devictor B, Bonnier P, Andrac L, Lavaut MN, Allasia C and Piana L (1992) Expression of HER-2 neu oncogene in breast cancer: correlation of quantitative immunochemistry and prognostic factors. *Int J Oncol* **1**: 815–823

Charpin C, Bonnier P, Devictor B, Andrac L, Lavaut MN, Allasia C and Piana L (1993) Immunodetection of Her-2/neu protein in frozen sections evaluated by image analysis: correlation with overall and disease-free survival in breast carcinomas. *Anticancer Res* **13**: 603–612

Charpin C, Vielh P, Duffaud F, Devictor B, Andrac L, Lavaut MN, Allasia C, Horschowski N and Piana L (1994) Quantitative immunocytochemical assays of P-glycoprotein in breast carcinomas: correlation to messenger RNA expression and to immunohistochemical prognostic indicators. *J Natl Cancer Inst* **86**: 1539–1545

Charpin C, Devictor B, Andrac L, Amabile J, Bergeret D, Lavaut MN, Allasia C and Piana L (1995*a*) p53 quantitative immunocytochemical analysis in breast carcinomas. *Hum Pathol* **26**: 159–166

Charpin C, Devictor B, Bergeret D, Andrac L, Boulat J, Horschowski N, Lavaut MN and Piana L (1995*b*) CD31 quantitative immunocytochemical assays in breast carcinomas. Correlation with current prognostic factors. *Am J Pathol* **103**: 443–448

Clark GM and McGuire WL (1991) Follow up study of HER-2/neu amplification in primary breast cancer. *Cancer Res* **51**: 944–948

Grogan TM, Casey TT, Miller PC, Rangel CS, Nunnery DW and Nagle RB (1993) Automation of immunohistochemistry. In *Advances in Pathology and Laboratory Medicine*, Mosby Year Book **6**: 253–283

Grogan TM, Rangel C, Rimsza L, Bellamy W, Martel R, McDaniel D, McGraw B, Richards W, Richter L, Rodgers P, Rybski J, Showalter W, Vela E and Zeheb R (1995) Kinetic-mode, automated double-labeled immunohistochemistry and in situ hybridization in diagnostic pathology. In *Advances in Pathology and Laboratory Medicine*, Mosby Year Book **8**: 79–99

Gusterson BA, Machin LG, Gullick NJ, Gibbs NM, Powles TJ, Elliot C, Ashley S, Monaghan P and Harrison S (1988) C erb B-2 expression in benign and malignant breast disease. *Br J Cancer* **58**: 453–457

Heatley M, Maxwell P, Whiteside C and Toner PG (1993) C erb B-2 oncogene product expression depends on tumour type and is related to oestrogen receptor and lymph node status in human breast carcinoma. *Pathol Res Pract* **189**: 261–266

Heintz NH, Leslie KO, Rogers LA and Howard PL (1990) Amplification of the c erb B-2 oncogene and prognosis of breast adenocarcinoma. *Arch Pathol Lab Med* **114**: 160–163

Kury F, Slintz G, Schemper M, Reiner G, Reiner A, Jakesz, Wrba F, Zeillinger R, Knogler W, Huber J, Holzner H and Spona J (1990) Her-2 oncogene amplification and overall survival of breast carcinoma patients. *Eur J Cancer* **26**: 946–949

Lipponen HJ, Aaltomaa S, Syrjanen S and Syrjanen K (1993) C erb B-2 oncogene related to p53 expression, cell proliferation and prognosis in breast cancer. *Anticancer Res* **13**: 1147–1152

McCann AH, Dervan PA, O'Regan M, Codd MB, Gullick WJ, Tobin BMJ and Carney DN (1991) Prognosis significance of c erb B-2 and estrogen receptor status in human breast cancer. *Cancer Res* **51**: 3296–3303

McGuire WL and Clark G (1992) Prognostic factors and treatment decisions in axillary node-negative breast cancer. *N Engl J Med* **326**: 1756–1760

Paterson MC, Dietrich KD, Danyluk J, Paterson AHG, Lees AW, Hanson NJJ, Jenkins H, Krause BE, McBlain WA, Slamon DJ and Fourney RM (1991) Correlation between C erb B-2 amplification and risk of recurrent disease in node negative breast cancer. *Cancer Res* **51**: 556–567

Pauletti G, Godolphin W, Press MF and Slamon DJ (1996) Detection and quantitation of HER-2/neu gene amplification in human breast cancer archival material using fluorescence in situ hybridization. *Oncogene* **13**: 63–72

Piffanelli A, Dittadi R, Catozzi L, Gion M, Capitanio G, Gelli MC, Brazzale A, Malagutti R, Pelizzola D, Menegon A, Giovannini G and Gardini G (1996) Determination of Erb B2 protein in breast cancer tissues by different methods. Relationships with other biological parameters. *Breast Cancer Res Treat* **37**: 267–276

Press MF, Pike MC, Chazin VR, Hung G, Udove JA, Markowicz M, Danyluk J, Godolphin W, Sliwkowski M, Akita R, Paterson M and Slamon DJ (1993) Her-2/neu expression in node-negative breast cancer: direct tissue quantitation by computerized image analysis and association of overexpression with increased risk of recurrent disease. *Cancer Res* **53**: 4960–4970

Quenel N, Wafflart J, Bonichon F, de Mascarel I, Trojani M, Durand M, Avril A and Coindre JM (1995) The prognostic value of c erb B-2 in primary breast carcinomas: a study on 942 cases. *Breast Cancer Res Treat* **35**: 283–291

Ravdin PM and Chamness GC (1995) The c erb B-2 proto-oncogene as a prognostic and predictive marker in breast cancer: a paradigm for the development of other macromolecular markers. *Gene* **159**: 19–27

Shou DJ, Ahuja H and Cline MJ (1989) Proto-oncogene abnormalities in human breast cancer. c erb B-2 amplification does not correlate with recurrence of disease. *Oncogene* **4**: 105–108

Slamon DJ, Clark GM, Wong SG, Levin WJ, Ullrich A and McGuire WL (1987) Human breast cancer: correlation of relapse and survival with amplification of the HER-2/neu oncogene. *Science* **235**: 177–182

Slamon DJ, Godolphin W, Jones LA, Holt JA, Wong SG, Keith DE, Levin WJ, Stuart SG, Udove J, Ullrich A and Press MF (1989) Studies of the Her-2/neu proto-oncogene in human breast and ovarian cancer. *Science* **244**: 707–712

Stal O, Sullivan S, Wingen S, Skoog L, Rutqvist LE, Cartensen JM and Nordenskjöld B (1995) C erb B-2 expression and benefit from adjuvant chemotherapy and radiotherapy of breast cancer. *Eur J Cancer* **31A**: 2185–2910

Tandon AK, Clark GM, Chamness GC, Ullrich A and McGuire WL (1989) Her-2/neu oncogene protein and prognosis in breast cancer. *J Clin Pathol* **7**: 1120–1128

Van de Vijver MJ, Peterse JL, Mooi WJ, Wisman P, Lomans J, Dalesio O and Nusse R (1988) Neu-protein overexpression in breast cancer. *N Engl J Med* **319**: 1239–1245

Walker RA, Gullick WJ and Varley JM (1989) An evaluation of immunoreactivity for c erb B-2 protein as a marker of poor short term prognosis in breast cancer. *Br J Cancer* **60**: 426–429

© Cancer Research Campaign 1997

Winstanley J, Cooke T, Murray GD, Platt-Higgins P, George WD, Holt S, Myskov M, Spedding A, Barraclough BR and Rudland PS (1991) The long term prognostic significance of c erb B-2 in primary breast cancer. *Br J Cancer* **63**: 447–450

Wright C, Angus B, Nicholson S, Sainsbury JRC, Cairns J, Gullick WL, Kelly P, Harris AL and Horne CHW (1989) Expression of c erb B-2 oncoprotein: a prognostic indicator expression in human breast carcinoma. *Br J Cancer* **49**: 2087–2090

*© Cancer Research Campaign 1997*